# Metabolic response to drought in six winter wheat genotypes

**DOI:** 10.1371/journal.pone.0212411

**Published:** 2019-02-19

**Authors:** Tihana Marček, Kamirán Áron Hamow, Balázs Végh, Tibor Janda, Eva Darko

**Affiliations:** 1 Department of Food and Nutrition Research, Faculty of Food Technology, Josip Juraj Strossmayer University of Osijek, Osijek, Croatia; 2 Department of Zoology, Plant Protection Institute, Centre for Agricultural Research of the Hungarian Academy of Sciences, Budapest, Hungary; 3 Department of Plant Physiology, Agricultural Institute, Centre for Agricultural Research of the Hungarian Academy of Sciences, Martonvásár, Hungary; National Research Council of Italy, ITALY

## Abstract

Wheat is one of the most important cereals, whose growth and development is strongly limited by drought. This study investigated the physiological and metabolic response of six winter wheat cultivars to drought with the emphasis on the induction of dominant metabolites affected by the treatment and genotypes or both. The plants were exposed to a moderate (non-lethal) drought stress, which was induced by withholding watering for six days under controlled greenhouse conditions. A decline in CO_2_ assimilation (Pn) and transpiration rate, stomata closure, a decrease in relative water content (RWC) and increase of malondialdehyde content were observed in drought-treated plants of all cultivars. These changes were most pronounced in Ellvis, while Soissons was able to retain the higher RWC and Pn. Among the studied metabolites, sugars (sucrose, glucose, fructose, several disaccharides), organic acids (malic acid, oxalic acids), amino acids (proline, threonine, gamma-aminobutyric acid (GABA), glutamine) and sugar alcohols such as myo-inositol accumulated to higher levels in the plants exposed to drought stress in comparison with the control. The accumulation of several metabolites in response to drought differed between the genotypes. Drought induced the production of sucrose, malic acid and oxalic acid, unknown organic acid 1, unknown disaccharide 1, 2 and 3, GABA, L-threonine, glutamic acid in four (Soissons, Žitarka, Antonija or Toborzó) out of six genotypes. In addition, Soissons, which was the most drought tolerant genotype, accumulated the highest amount of unknown disaccharide 5, galactonic and phosphoric acids. The two most drought sensitive cultivars, Srpanjka and Ellvis, demonstrated different metabolic adjustment in response to the stress treatment. Srpanjka responded to drought by increasing the amount of glucose and fructose originated from hydrolyses of sucrose and accumulating unidentified sugar alcohols 1 and 2. In Ellvis, drought caused inhibition of photosynthetic carbon metabolism, as evidence by the decreased Pn, gs, RWC and accumulation levels of sugar metabolites (sucrose, glucose and fructose). The results revealed the differences in metabolic response to drought among the genotypes, which drew attention on metabolites related with general response and on those metabolites which are part of specific response that may play an important role in drought tolerance.

## Introduction

Wheat (*Triticum aestivum* L.) is one of the most important cereals used in daily human consumption as a main source of proteins and carbohydrates. Its growth, development and yield are strongly limited by drought that causes huge economic losses worldwide, even in the non-wheat producing areas like South and Central Asia region [1, 2]. It is estimated that between 2005 and 2015 the drought caused 30% crop damage in developing countries of Latin America, Asia, Africa and Pacific Islands, which amounted to over 29 billions of dollars in loss making drought as the most expensive natural disaster [3]. Since drought periods are predicted to become more frequent and severe due to accelerated climate changes, the studies on wheat response to drought become more and more acute [4].

Drought occurs when available water is not sufficient for optimal growth and development of plants [5]. Plants sense drought when water provision of the root system becomes restricted or when the transpiration rate becomes so intense that it causes imbalance between water intake and water loss [6]. At the beginning of the drought, plants usually close the stomata, which limit the carbon uptake of the leaves, with concomitant decrease of CO_2_ assimilation rate (Pn) and intercellular CO_2_ level [6, 7]. Besides the stomatal closure–especially during long, severe drought–CO_2_ assimilation is also inhibited by several non-stomatal events, which results in a decrease of ATP production [8] and several metabolic failures [6, 7, 9]. The drought-induced decline of photosynthesis relates to the changes of plant metabolic responses including sugars and starch production, to the accumulation of compatible osmolites and activation of abscisic acid (ABA) dependent and independent regulatory pathways [10, 11, 12]. In addition, the inhibition of the photosynthetic CO_2_ assimilation results in an imbalance between light absorption and its usage, thus leading to the development of oxidative stress conditions [13]. Accumulation of reactive oxygen species (ROS) also affect the metabolic response of plants.

The responses of many plant species to drought stress have been extensively studied and several primary and secondary metabolites have been identified that take part in drought stress response [14, 15, 16]. Proline (Pro) and quaternary ammonium compound glycine-betaine (GB) are often accumulated during drought [17]. They have osmoprotective role in the maintenance of cell turgidity. While reducing the osmotic potential of the cells, these compounds both retain the cellular water content and stabilize the cellular structures by forming hydration shell around the proteins [18, 19]. Pro and GB also have a protective role against ROS, provide stability to the macromolecules (such as lipids, nucleic acids, proteins) and may act as sources of nitrogen and carbon under drought [20, 21]. Other amino acids, like serine or GABA are also reported to accumulate under drought stress [22, 23]. Drought can also trigger the accumulation of soluble sugars (both mono- and disaccharides), sugar alcohols, such as fructans, myo-inositol and mannitol [24, 25]. They preserve the structure of functional and structural proteins of the cell in a manner that promote the stabilization of the subunits against the water loss [26, 27]. Moreover, several osmolites including sucrose, glucose and fructose can serve as metabolic precursors in different biosynthetic pathways and together with GABA they can act as signalling molecules that control the expression of several genes involved in different metabolic pathways [28–30].

Metabolic adjustment in response to drought is a dynamic and multifaceted process which depends on the strength and duration of drought and also on the sensitivity of cultivars. Traditional metabolic studies focused on single metabolites or groups of metabolites. Today, the modern analytical techniques such as gas chromatography mass spectrometry (GC-MS) provide powerful tools for tracking the metabolomics alternations occurring in plants during water deficit. The GC-MS analyses allow two different approaches for identification of metabolite: non-targeted and targeted. The non-targeted metabolic analyses provide a comprehensive insight in alternations of all the measurable metabolites in the samples. It is not as sensitive as the selective, targeted metabolomics that focuses on some special metabolite groups [31], but it enables to demonstrate the complexity of metabolic adjustment to drought and reveal the different responses of genotypes to drought [32, 33].

In the present study, the drought-induced changes in leaf photosynthesis, loss of water, osmotic potential and metabolic adjustment were compared in six winter wheat genotypes. Our aim was to present differences in the metabolites that play important role in metabolic and physiological responses to drought. Particular question was to find which physiological and metabolic changes were general in these genotypes and which ones depends on the genotype. Analyses of metabolite pools found in leaf saps can help to understand the drought tolerance mechanisms and selection of wheat varieties tolerant to drought.

## Material and methods

### Plant material, growth conditions and drough treatment

The experiment was carried out on six winter wheat (*Triticum aestivum* L.) genotypes Soissons, Ellvis, Mv. Toborzó, Srpanjka, Antonija and Žitarka. The surface-sterilized seeds were germinated on wet filter paper in Petri dishes for 3 days at room temperature, and planted into Jiffy-7 pellets (www.jiffygroup.com). The 5-day-old seedlings were vernalized at 4°C for 6 weeks under low photosynthetic photon flux density (PPFD) (20 μmolm^-2^s^-1^). After that, plants were grown in 2L pots (1 plant/pot) filled with a 3:2:1 mixture of garden soil, compost and sand in a plant growth chamber (PGV-36, Conviron, Controlled Environments Ltd., Winnipeg, Canada) for 4 weeks under the following day/night conditions: 16/8 h day/night photoperiod at 22/18°C day/night temperature, 250 μmol m^-2^ s^-1^ PPFD, and 75% relative humidity (RH). Afterwards, 20 plants of each genotype were placed in a greenhouse (Global Glasshouse Venlo) in randomized complete block design. The growth conditions were similar for all plants till flag leaf sheath extending stage (Z41 stage of Zadoks’s scale [34]). The average day/night temperature were 30/22°C and maximum light intensity was 750 μmolm^-2^s^-1^. The plants were irrigated regularly keeping the volumetric soil moisture content (VSMC) values between 30 and 35%, where VSMC was measured by an HH2 moisture meter (Delta T device SM -100 sensor, Delta-T Devices Ltd, Cambridge, UK Delta-T Devices Ltd., Cambridge, UK).

After the plants reached the flag leaf sheath extending developmental stage (Z41 stage of Zadoks’s scale), the pots of each genotype were divided into two sets. One set represented the control plants and the second the drought-treated plants. Each set comprised eight to ten pots per genotype. Drought stress was induced by withholding water and the average daily VSMC was kept between 10 and 15% during the drought treatment. This VSMC represented a non-lethal, moderate drought stress. It was chosen to activate the drought-induced metabolic responses. The average VSMC values were kept continuously between 30 and 35% in case of the control plants.

### Determination of the photosynthetic activity of leaves by gas exchange measurement

The photosynthetic activity of control and drought-treated plants were determined on intact attached leaves after 6 days of drought treatment. The measurements were performed on five randomly selected fully developed leaves with a Ciras 3 Portable Photosynthesis System (PP Systems, Amesbury, MA, USA) using a narrow (1.7 cm^2^) leaf chamber, similarly as described in [35]. The net photosynthetic rate (Pn), stomatal conductance (gs), transpiration rate (E) and internal CO_2_ concentration (Ci) were determined at the steady state level of photosynthesis using a CO_2_ level of 390 μL L^-1^ and light intensity of 500 μmol m^-2^ s^-1^.

### Sample collection

Leaf samples were collected from control and drought-treated plants after 6 days of treatment for determination of relative water content (RWC) and malondialdehyde (MDA) contents of leaves and for isolation of leaf sap. In this latter case, samples were immediately frozen in liquid nitrogen and stored at -80°C prior to analysis.

### Determination of RWC and MDA contents of leaves

RWC content of leaves were determined in the middle part of the fully expanded leaves collected from control and drought-treated plants after 6 days of treatment. Five, 2 cm long leaf segments (app. 200 mg) were used for each sample and four biological repetitions were used for each treatment (control and drought treatment). RWC was determined by measuring the fresh weight (FW) of leaf segments, saturated weight (SW) after 24 h rehydration on distilled water at 4°C in the dark, and dry weight (DW) after oven drying for 48 h at 80°C. The RWC was calculated as the percentage of the amount of water in the leaf tissue at sampling to the amount of water present at fully turgid state as described in [36].

The MDA content of samples (300 mg fresh leaves per sample) was determined as described by Batish et al. [37] and five biological repetitions were used for the measurements.

### Isolation of leaf sap and determination of osmotic potential of leaf tissue

Leaf saps were isolated for determination of the osmotic potential of leaves and the concentration of metabolites in leaf tissue. One gram of leaves was crushed in liquid nitrogen and the cold powders were transferred into centrifuge tubes containing micro-SpinFilter (Micro-SpinFilter Tubes, Fisher Scientific; 0.45 μm) and centrifuged at 10 000 rpm for 10 min at 4°C. The leaf saps were divided to aliquots before storing them at -80°C for different measurements. At least 3 isolation processes were performed for each genotype and treatment.

The osmotic potential of leaf saps was determined using a freezing point osmometer (Osmomat 030, Gonotech, Germany) and the osmotic potential (ψ_π_) values were calculated according to Bajji et al. [38].

### Determination of proline and glycine-betaine concentration of leaf sap

The proline and GB contents were determined from leaf saps diluted (1:17.5) with ultrapure water. The determination of proline content was based on its reaction with ninhydrin and GB was measured by using the periodide method according to Bates et al. [39] and Grieve and Grattan [40], respectively.

### Determination of polar metabolites from the leaf sap by GC-MS

Before the extraction of polar metabolites, 60 μl of leaf sap of each sample was diluted with 140 μl of ultrapure water. The samples were boiled and centrifuged at 10000 g for 10 min. An aliquot of 100 μl of supernatant was completed with 6 μg ribitol (as an internal standard) and these samples were used for the extraction.

Sample extraction and GC-MS analysis was carried out according to Schauer et al. [41] with slight modifications as described by Juhász et al. [42]. The method is based on extraction with methanol and water followed by separation of polar compounds by adding chloroform, and then the methanol/water layer was evaporated under vacuum. The dried extracts were methoxyaminated with methoxyamine hydrochloride (MEOX) in pyridine and derivatized with N-methyl-N-(trimethylsilyl) trifluoroacetamide (MSTFA). Samples were analyzed in split mode in a GCMS-QP2010 system (Shimadzu, Kyoto, Japan) where chromatography was carried out on an SLB-5ms 30m × 0,25mm × 0,25μm capillary column. Analysis conditions were the same as described by Juhász et al. [42]. Mass spectra were recorded at 5 scans sec^-1^ with an m/z 45–650 scanning range. Data analysis was carried out using Shimadzu GC-MS Solution Postrun analysis software with searching the Wiley 9^th^ edition mass spectral database and comparing the analytes using the Kovats retention index (RI). For quantification, all peak areas were normalized to the response of ribitol. In addition, the sugars and amino acids, myo-inositol and some organic acids were identified and quantified based on a comparison of the retention time and mass spectrum to an authentic standard that was analysed under identical conditions. Where a reference material was unavailable, amounts were estimated by the use of a standard from the same compound class having the closest tentative molecular weight. Capillary column, all chemicals and standards were purchased from Sigma-Aldrich (Darmstadt, Germany). The method and protocol has been verified for leaf sap matrix where average RSD% for the response of 20 analytes selected from all analyzed compound classes was less than 5% for five technical replicates.

### Statistical analyses

Eight to 10 plants of each genotype and treatments were used in the experiments. Randomized complete block design was used both in the phytotron growth chamber and in the greenhouse in order to minimize the differences caused by the environment. Samples were collected from each pot and the measurements were performed in 3–5 biological replicates per genotypes and treatments. Factorial analysis of variance (ANOVA) was conducted using STATISTICA software package (version 13.4) for determination of the effect of genotypes (G), drought treatment (T) and G × T. In addition, Tukey’s *post hoc* test was also used for the determination of the statistical significant difference between the mean values. The correlation between the measured physiological parameters and metabolites were also determined with Spearmanʾs rank order correlation coefficients (R). Principal component analysis (PCA) was used to evaluate and discriminate the metabolic response of different wheat genotypes subjected to drought and well-watered conditions. The data set used for PCA consisted of 32 variables. PCA was applied to the standardized data set and the factor loadings were done in order to estimate the proportion of total variance with different principal components. The loadings showed correlations among different principal components (PC) and measurable variables whereby high loadings represented strong correlation (>0.75) [43].

## Results

### Effect of drought on RWC, photosynthetic parameters and MDA content of leaves

**Fig 1:**
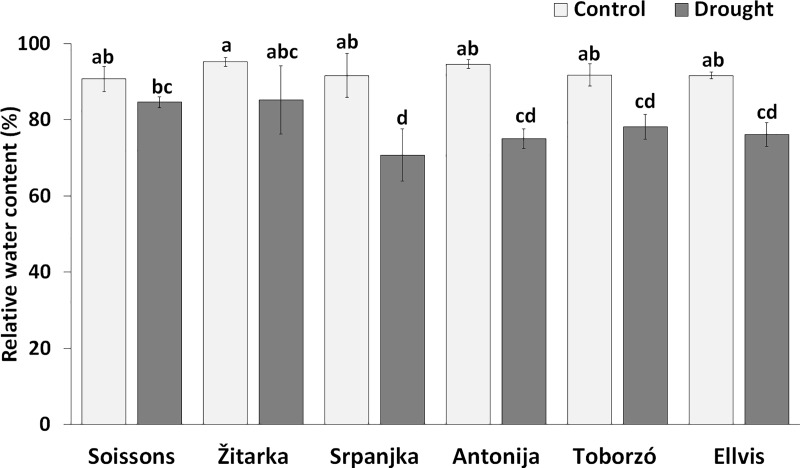
The relative water content (RWC) in wheat genotypes under control and drought conditions. Data are means ± SD of four replicates per treatment and genotype. Different letters indicate significant differences among the mean values at P < 0.05 using Tukey’s *post hoc* test.

The RWC contents were similar under well-watered conditions in all genotypes. Drought stress resulted in a decrease of RWC content in most genotypes except in wheat Soissons and Žitarka. In these genotypes the decreases were not statistically significant as compared to control plants (Fig 1). The lowest RWC content of leaves was found in Srpanjka. This genotype showed lower ability to reserve water as compared with Žitarka and Soissons.

The drought-induced stomatal closure and inhibition of CO_2_ assimilation were monitored by gas exchange measurements (Table 1). All wheat genotypes showed similar photosynthetic properties in control plants as indicated by the similar values of CO_2_ assimilation rate (Pn), the stomatal conductance (gs), transpiration rate (E), and intercellular CO_2_ level (Ci) among the genotypes. These parameters decreased significantly under drought in all genotypes (Table 1), which indicates a decrease in CO_2_ assimilation rate, the stomatal closure and a decrease of transpiration to avoid the water loss of plants. Comparing the genotypes, the most pronounced decrease of Pn, gs and E was found in Ellvis followed by Srpanjka, while the highest values of these parameters were detected in Soissons (Table 1).

**Table 1:** The photosynthesis rate (Pn), stomatal conductance (gs), intercellular CO_2_ level (Ci) and transpiration rate (E) in wheat genotypes.

	Pn (A)		gs		Ci		E	
Genotypes	Control	Drought	Control	Drought	Control	Drought	Control	Drought
Soissons	13.4±0.6**a**	8.3±0.9**b**	304±15A	114±13B	282±4*a*	238±18*b*	3.5±0.26a	1.3±0.10b
Žitarka	13.7±0.5**a**	6.0±1.2**cd**	297±13A	68±9C	276±3*a*	221±18*b*	3.5±0.18a	0.9±0.09c
Srpanjka	14.1±0.2**a**	5.5±1.0**cd**	291±14A	57±9CD	277±5*a*	229±24*b*	3.5±0.15a	0.6±0.11c
Antonija	13.9±0.3**a**	6.4±1.3**c**	286±13A	72±12C	274±6*a*	240±5*b*	3.4±0.11a	0.9±0.23c
Toborzó	14.3±0.4**a**	6.8±0.6b**c**	286±18A	65±15CD	281±9*a*	229±30*b*	3.4±0.27a	0.78±0.19c
Ellvis	14.4±0.4**a**	4.3±1.2**d**	295±6.2A	40±4D	275±4*a*	227±9b	3.5±0.15a	0.58±0.08c

Values are means ± S.D. (n = 5). Tukey’s *post hoc* test was used to compare the mean values of genotypes within each measured parameter. The different letters indicate statistically significant differences at P<0.05.

**Fig 2:**
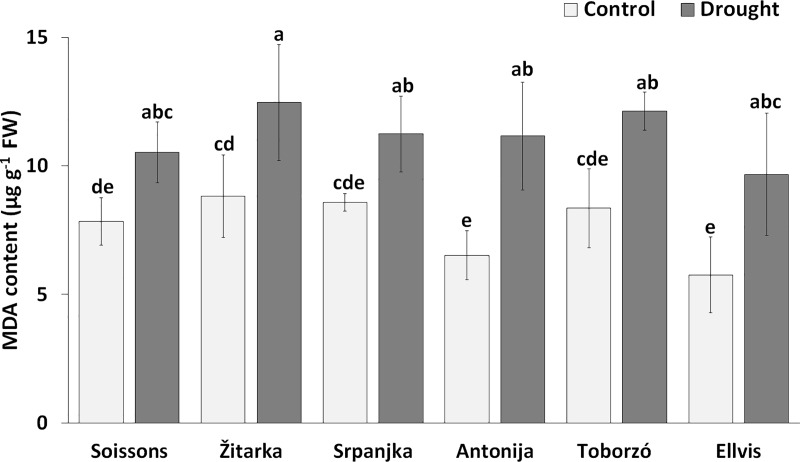
The MDA content in wheat genotypes under control and drought conditions. Values are means ± S.D. (n = 5). Different letters indicate significant differences among the mean values of genotypes at P < 0.05 using Tukey’s *post hoc* test.

MDA content indicates the membrane damage through lipid peroxidation. Lower MDA content was detected in all genotypes under control conditions, while the MDA content increased under drought stress (Fig 2). However, there was no significant difference in MDA content among the genotypes under drought stress conditions.

Comparative statistical analyses were performed to indicate the interactions of all physiological factors (gas exchange parameters, RWC, and MDA content). The results are presented in S1 Table. The drought stress (treatment, T) affected all parameters, while genotypic variations were found in the drought stress response of most of the traits with the exception of Ci and MDA content. The significant effects for G × T were noticed in the cases of RWC, Pn, gs and E.

### Effect of drought on osmotic potential, proline and glycine-betaine contents

**Fig 3:**
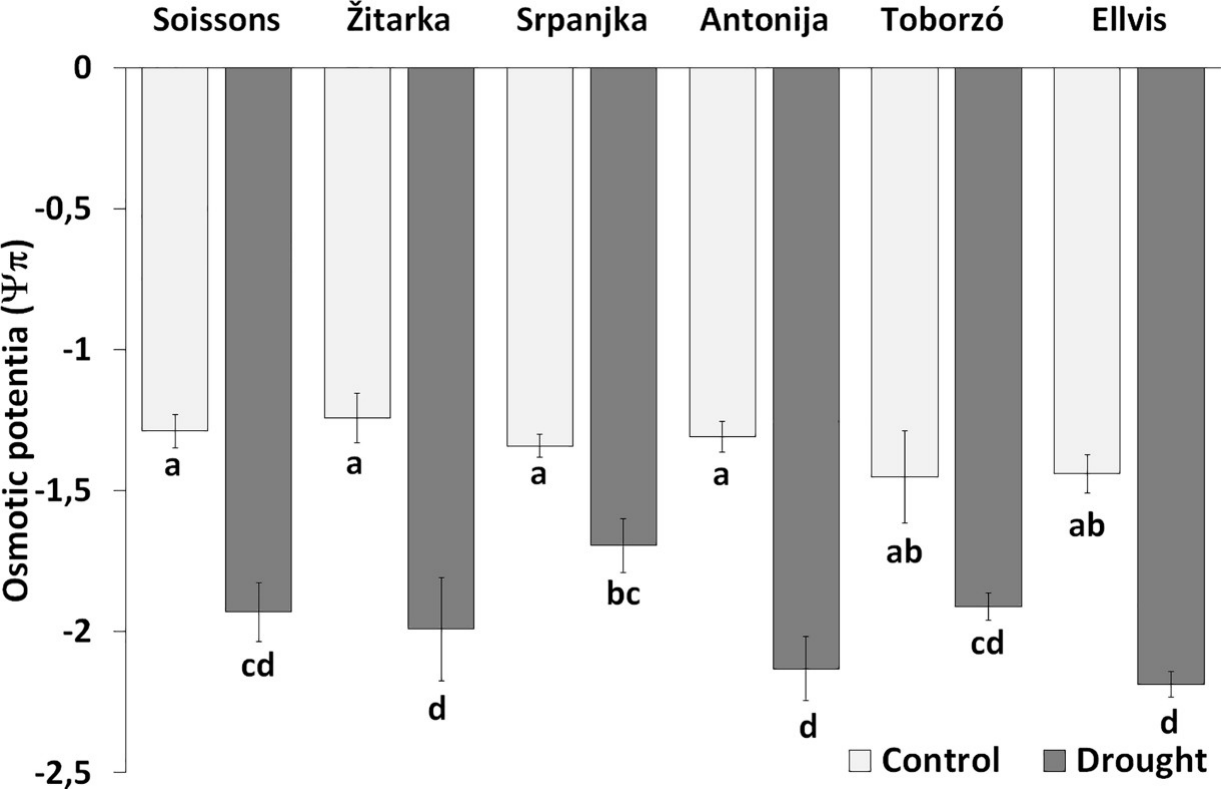
The osmotic potential (ψ_π_) of leaf saps of wheat genotypes grown under control and drought conditions. Values are means ± S.D. (n = 3). The different letters indicate statistically significant differences among the mean values of genotypes at P < 0.05 using Tukey’s *post hoc* test.

The osmotic potential (ψ_π_) of leaf sap decreased under drought stress (Fig 3). The decrease was less pronounced (appr. 30%) in genotypes Srpanjka than in Antonija, Žitarka and Ellvis, where the decrease ranged between 50 and 60%. Considering the drought-induced changes in the osmotic potential and RWC together, it seems that the decrease of ψ_π_ was mainly due to the water loss of leaves in Srpanjka, Ellvis, and Antonija. No similar correlation was found between these two parameters in Soissons and Žitarka, indicating that other factors may contribute to the decrease of ψ_π_ in these genotypes.

To determine which compatible solutes can be responsible for the decrease of osmotic potential, the amount of several compatible solutes were determined from the leaf saps.

**Fig 4:**
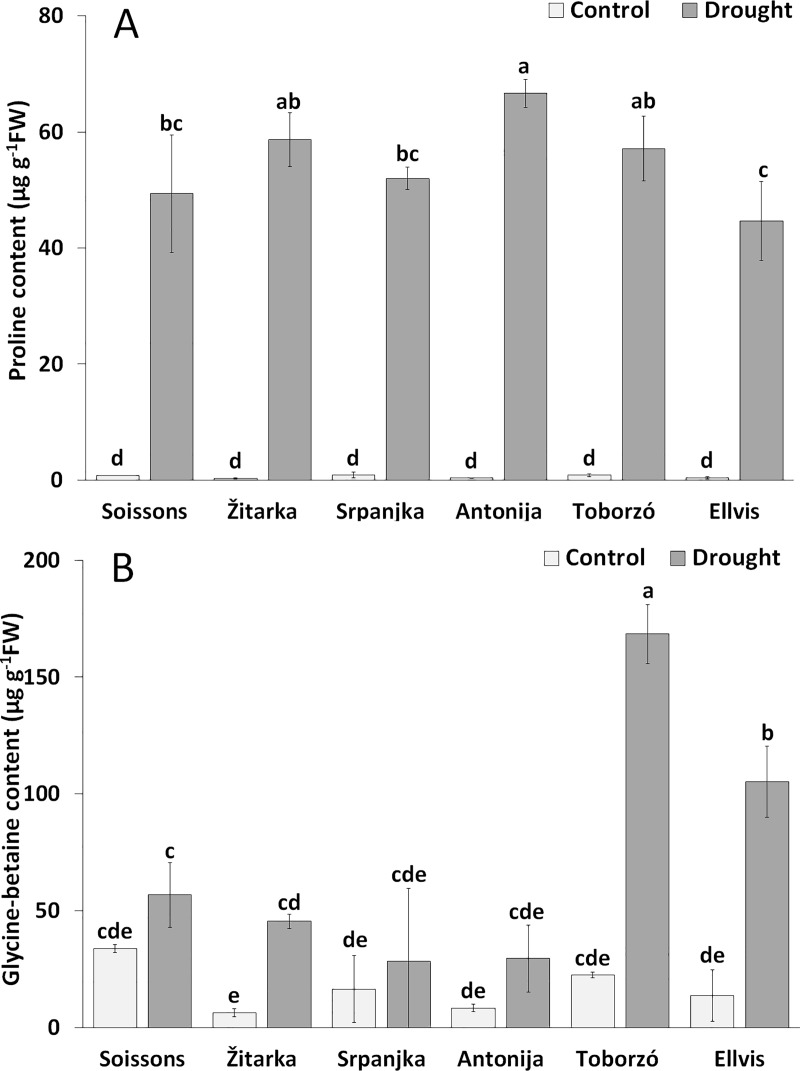
**The changes of proline (A) and glycine betaine (B) contents of leaf saps of wheat genotypes grown under control and drought conditions.** Values are means ± S.D. (n = 3). The different letters indicate statistically significant differences among the mean values of genotypes at P < 0.05 using Tukey’s *post hoc* test.

The contents of proline remarkably increased under drought stress in all genotypes as compared with the corresponding controls (Fig 4A). The highest proline content was detected in Antonija followed by Žitarka, Toborzó, Srpanjka and Soissons, while the lowest proline content was observed in Ellvis.

The increment of GB content was less intense than it was for proline; it ranged between 1.7-fold (in Srpanjka and Soissons) and 7.7-fold (in Ellvis). The highest GB content was found in wheat Toborzó followed by Ellvis (Fig 4B).

To indicate connection between the osmotic potential and the compatible solutes, proline and GB, analysis of variance was performed (S2 Table). Drought stress (treatment, T) and genotype (G) had significant impact on all parameters, which can be seen through genotype (G) and treatment (T) interactions (G × T) too suggesting similar drought response.

### Effect of drought on the accumulation of other polar metabolites

**Fig 5:**
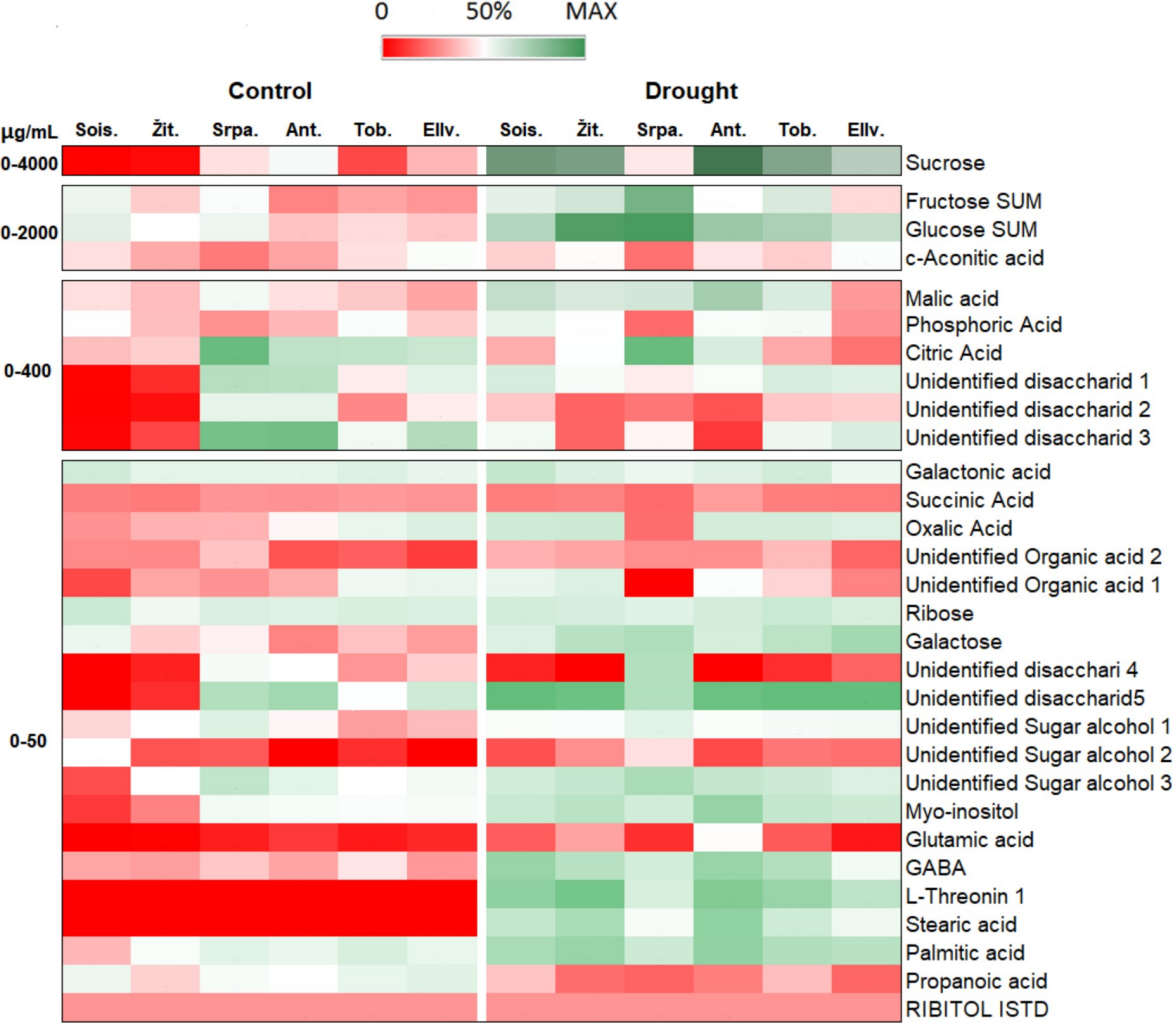
Heat map presenting the metabolite accumulation under control and drought conditions in wheat genotypes Soissons, Žitarka, Srpanjka, Antonija, Toborzó and Ellvis. Values are means ± S.D. of three replicates per treatment and genotype. Different colour represents different concentration of metabolites. Abbreviations in the heat map means: Sois. (Soissons), Srpa. (Srpanjka), Žit. (Žitarka), Ant. (Antonija), Tob. (Toborzó) and Ellv. (Ellvis), respectively.

The composition of polar primary metabolites was also determined from the leaf saps of the six wheat genotypes under control and drought conditions using GC-MS analyses. In total 30 metabolites were detected from the leaf saps and 20 of them were identified. These metabolites are distributed into 5 classes, such as sugars, sugar alcohols, organic acids, lipids and amino acids and presented in S3 Table. To visualise the comparison of genotypes and treatments, the amount of the polar primary metabolites was also presented in a heat-map (Fig 5). The results of the comparative statistical analyses which indicated the interactions of all metabolic parameters (sugar components, lipids, organic acids, amino acids and sugar alcohols) are presented in S4–S6 Tables.

Leaf saps contained sugars (such as sucrose, fructose and glucose) in the largest amount. Regarding content, they were followed by cis-aconite acids and malic acids, the two compounds of citrate cycle (TCA cycle). The amount of phosphoric acids, citric acids and several unidentified disaccharides (1–3) ranged between 0 and 400 μg/mL, while several minor compounds ranged between 0 and 50 μg/mL were also determined in the leaf saps (Fig 5).

Generally, the concentration of many polar primary metabolites significantly increased under drought exposure in regards to control in most of the genotypes. Active metabolism response to drought was observed for 7 sugar components (sucrose, glucose, fructose, galactose and unidentified disaccharides 1, 2, 3 and 5), 2 fatty (stearic and palmitic) acids, 3 organic acids (malic and oxalic acid, unidentified organic acid 2), 2 sugar alcohols (myo-inositol and unidentified alcohols 3) and 3 amino acids (GABA, glutamic acid and L-threonine) (Fig 5). The amount of 7 metabolites (ribose, galactonic acid, succinic acid, cis-aconitic acid, phosphoric acid and unidentified organic acids 1) remained unchanged under drought application as compared to control. Suppressed metabolism response was found for unidentified disaccharides 4 and propanoic acid under drought stress (Fig 5 and S3 Table).

Besides these general responses, these changes were not always uniform among the genotypes. Significantly higher amount of sucrose was found in wheat Soissons, Antonija and Žitarka than in Ellvis and Srpanjka (S3 Table) under drought stress conditions. For instance, the values of sucrose in Antonija was 3.3 times higher than in Srpanjka, which exhibited the lowest amount of this sugar. However, in wheat Srpanjka the low amount of sucrose was associated with high amount of fructose and glucose. This connection was not observed in other genotypes. The total amount of these three sugar compounds (sucrose, fructose and glucose) was the lowest in wheat Ellvis. Drought caused accumulation of galactose in most of the genotypes except in Soissons. The difference in galactose content was most pronounced between Ellvis and Soissons (S3 Table). Compared with Soissons, Ellvis had 78% more galactose. Diverse sugar metabolite pattern response of genotypes could also be observed in the unidentified disaccharides 1–3 and 5. While under control conditions, their level was low in Soissons and Žitarka and relatively high in Srpanjka and Antonija, their amount changed inversely under drought stress: namely, they increased significantly in Soissons and Žitarka and decrease in Srpanjka and Antonija. These compounds slightly changed in Toborzó and Ellvis (Fig 5). These results indicate that these disaccharides are the compounds of the same metabolic pathways, whose activation may differ among the genotypes. On the other hand, the six wheat genotypes differed in sugar metabolites under optimal water conditions too. In general, among all genotypes, Antonija and Srpanjka contained the higher amount of sucrose and all unidentified disaccharides (1–5). Significant accumulation of disaccharides (2, 3 and 4) was also recorded in Ellvis. Contrarily, Soissons displayed high amount of monosaccharides, glucose, galactose and fructose (Fig 5).

Besides the compounds of TCA-*cycle* (malic, citric, succinic, oxalic acids and cis-aconitic acids), phosphoric acids and galactonic acid (derived from galactose) were identified in the leaf saps (Fig 5 and S3 Table). Among the genotypes, differences in organic acids were apparent in the cases of citric and cis-aconitic acids under both drought and well-watered conditions (S3 Table). The lowest amount of cis-aconitic acid was detected in Srpanjka, while the amount of citric acid was extremely high in this genotype both under control and drought conditions. Drought stress induced an accumulation of the malic and oxalic acids of leaf saps in four genotypes (Soissons, Žitarka, Antonija and Toborzó), while they did not change significantly in Srpanjka and Ellvis. As a result of the drought stress, Soissons, Žitarka, Antonija and Toborzó accumulated significantly more unidentified organic acid 2 than Ellvis or Srpanjka (Fig 5). According to its mass spectra and retention index (RI), this organic acid is most likely to be maleic acid, the cis-isomer of fumaric acid.

Considering sugar alcohol metabolites, the myo-inositol concentration increased under drought stress in most of the genotypes, providing the highest values in Antonija and lowest in Srpanjka (Fig 5). In addition, drought-exposed plants contained higher amount of unidentified sugar alcohol 1 (which is most likely erythritol) in Ellvis and Toborzó, sugar alcohol 2 (most likely arabitol according to its mass spectra and RI) in the most genotypes except Soissons and sugar alcohol 3 in Žitarka, Toborzó and Soissons (S3 Table). Unexpectedly, in Soissons lower amount of unidentified alcohol 2 was recorded in drought treated samples than in the controls. Elevated concentration of sugar alcohol 1 in control plants was evident in Srpanjka, which produced (from 0.8 to 1.9 times) higher sugar alcohol 1 than the others genotypes.

Under control conditions there were no differences in the amount of free amino acids among the various genotypes (Fig 5 and S3 Table). Drought stress induced an accumulation of all detected amino acids, especially in GABA and L-threonine (Fig 5). The increases were more pronounced in Soissons, Žitarka, Antonija and Toborzó than in Ellvis or Srpanjka. Thus, significantly higher GABA, glutamic acid and L-threonine production was detected in Soissons, Žitarka, Antonija and Toborzó than in Ellvis or Srpanjka. For instance, the L-threonine level was 2 times higher in Žitarka than in Srpanjka under drought (S3 Table). The amount of glutamic acid was also increased by the stress conditions in the four genotypes, but not in Ellvis or Srpanjka.

The amount of free fatty acids, such as palmitic and stearic acids, also increased as a result of drought in all genotypes. Comparing the genotypes, the amount of stearic acid was significantly higher in Antonija, Žitarka, Toborzó and Soissons than in Srpanjka and Ellvis under drought stress conditions, in spite of the fact that there were no differences among the genotypes under well-watered conditions, where low amount of stearic acids was detected. Similarly the palmitic acid was also higher in those four genotypes (Antonija, Žitarka, Toborzó and Soissons) than in Srpanjka. The propanoic acid concentration declined under drought in all genotypes, especially in Ellvis and Srpanjka.

Analysis of variance of metabolic components displayed strong interactions among treatments (T) except in case of ribose, unknown disaccharide 1 (D1), cis-aconitic acid, phosphoric acid and unidentified organic acid 1 (OA1) (S3–S6 Tables). It seemed that the amount of these compounds was constant regardless of treatment. However, significant genotypic variation (G) was found for most of the compounds with exception of ribose and succinic acid. Similarly, considering genotype (G) × treatment (T) interaction, significances were found for most primarily metabolites except for ribose sugar, propanoic acid and several organic acids (phosphoric, galactonic, *cis*-aconitic and succinic).

Comparative statistical analyses were also performed to reveal the relationship between the osmotic potential and the changes of metabolites found in leaf saps (S7 Table). The osmotic potential was correlated positively to the amount of the glucose, galactose, sucrose, disaccharide 5, cis-aconite acid, malic acid, SA3, myo-inositol, the amount of glutamic acid, GABA threonine, GB and proline contents, and also to the amount of stearic and palmitic acids. Negative correlation was found for disaccharide 4, the amount of citric and succinic acids and propanoid acid, indicating that their amount decreased in parallel to the increase of osmotic potential (S7 Table).

### PCA analyses

**Fig 6:**
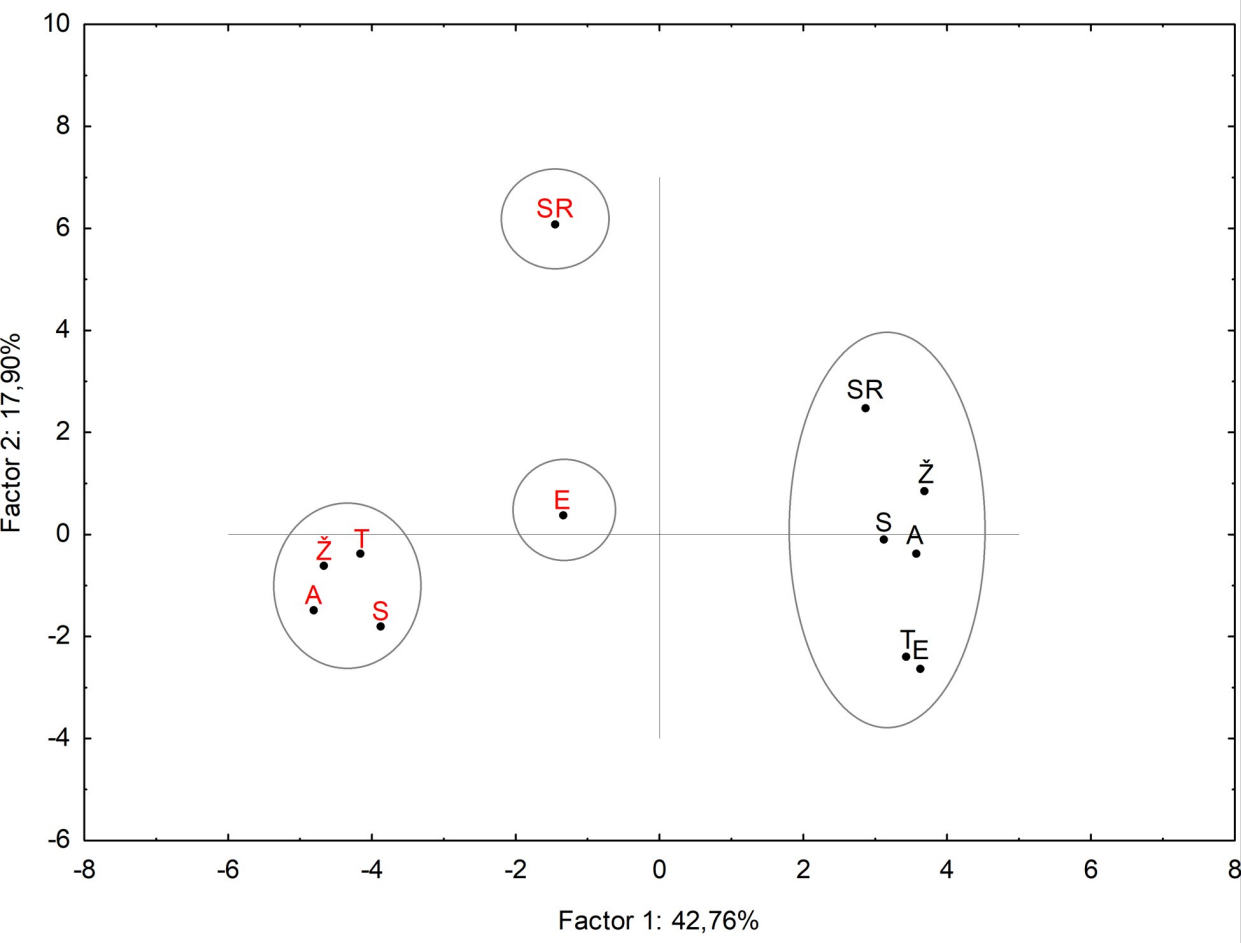
Principal component analysis of combined wheat genotypes data sets. Scores of the first two factors. Black-coloured letters indicate control; red-coloured letters indicate drought. Letters in the clusters means: S (Soissons), SR (Srpanjka), Ž (Žitarka), A (Antonija), T (Toborzó) and (Ellvis), respectively. Principle component analysis (PCA) was performed to discriminate the metabolic response of wheat genotypes. Data were analysed by using STATISTICA 13.4 software package.

S8 Table shows the percentage of total variance described by several principal components (PC) and their correlation with the metabolic compounds, proline, GB and osmotic potential. The PCA yielded the total variation of three principal components showing 78.07% of data variation under control and drought treatments (S8 Table). The most important were two components explaining 61% of data variance (S1 Fig). The first component (PC1) was largely determined by high negative loadings on myo-inositol, glutamic acid, GABA, L-threonine, stearic and palmitic acids, malic acid, sucrose, glucose, galactose, D5, GB and proline, while propanoic acid had high positive loadings. The second component (PC2) was largely determined by high positive loadings related to cis-aconitic and phosphoric acids and OA1, respectively. In the same component (PC2), strong negative loadings were recorded for fructose and SA1. Undetermined disaccharides (D1-D4) had high negative loadings in the third component (PC3). The score plot (Fig 6) showed four clusters. Cluster I separates control plants of six wheat genotypes grouped together based on metabolic response, which corresponds to well-water conditions (described as low amount of primarly metabolites, proline and GB contents or low Ψπ values). The variation found among the genotypes was due to the different amounts of sucrose, citric acid, unknown unidentified disaccharides (1–5) and sugar alcohols. However, these differences were less pronounced than those found under drought stress condition, therefore all genotypes were grouped together and separated from the drought-treated plants (Fig 6).

Drought treated genotypes of Toborzo, Žitarka, Soissons and Antonija were grouped into a cluster II due to similar metabolic pattern related to active metabolism pathways and osmotic adjustment accompanied with high production of compatible sugars and sugar alcohols. Ellvis and Srpanjka responded to drought differently. They showed similar response in OA1, L-threonine, proline content and palmitic and stearic acid, while based on some differences, the two genotypes were separated into various groups. Ellvis exhibited decreased oxalic and malic acids content and GABA production, and higher galactose level, cis-aconitic acid and GB amount, which classified it in cluster III. Cluster IV included Srpanjka with increased amounts of sugars (fructose and glucose), sugar alcohol 2 (SA2) and citric acid and lower oxalic acid.

## Discussion

### Drought stress response of the six wheat genotypes

The comparison of the drought stress responses of the six winter wheat genotypes revealed that the wheat Ellvis and Srpanjka showed intense water loss, while the wheat Žitarka and Soissons were able to preserve high RWC even under water limited conditions (Fig 1). The relationship between high RWC and drought tolerance has already been reported for various wheat genotypes [44–46]. Retention of water under adverse environmental condition could be a desirable trait, however, when the plants keep the stomata closed and the transpiration is low, little CO_2_ is taken up. In addition, low transpiration due to stomata closure also means poor cooling of the leaves and less uptake and transport of nutrients [47], which can adversely affect the plant metabolism. On the other hand, only sustained photosynthesis results in a better yield [48]. Finding the balance between maintenance of water content and photosynthetic activity of leaves is a big challenge for the plants. In a drought-tolerant wheat cultivar Changhan 58 lower reduction of Pn was observed after exposure to drought than in drought-sensitive cultivar Xinong 9871 [49], similarly as it was found for wheat Soissons. However, Changhan 58 showed lower transpiration rate than Xinong 9871, which is opposite to the drought response of Soissons in our study. The higher RWC accompanied with better photosynthetic activity, more open stomata and higher transpiration rate indicated occurrence of active metabolic re-arrangements in wheat Soissons, which enabled the plants to maintain normal cellular function and growth. The other genotypes showed more intense water loss, decrease of CO_2_ assimilation and stomatal closure than wheat Soissons (Table 1). These effects were most pronounced in wheat Ellvis. Altogether, both of the drought-induced cell dehydration (passive water loss) and the synthesis of osmotically active substances (called active osmotic adjustments) in the cells can lead to a decrease of the osmotic potential and an increase of the concentration of metabolites in the cells [50]. Therefore, it is difficult to determine which metabolic changes are involved in the processes of the passive water loss or active osmotic adjustments, and which metabolites provide common responses or are regulated in genotype dependent manner. We believe that the accumulation of different metabolites may reflect the differences in plant adaptation to drought. Therefore, we payed attention on those the metabolic changes which had essential role in the drought-stress response of genotypes and segregated in a similar manner as found by PCA analyses (Fig 6).

### Role of proline and GB in response to drought of the six wheat genotypes

Several investigations on maize [51] and wheat cultivars [52] have demonstrated that drought stimulate proline and GB production and that these compatible solutes are important in drought tolerance [53, 54]. In the present experiments, the accumulation of proline and GB was also observed, however, their changes did not refer to the observed physiological parameter and differences in the adaptation to stress. Namely, similar proline content was found in Soissons, Srpanjka and Ellvis, and GB content in Toborzó and Ellvis (Fig 4A). These results suggest that although proline and GB have osmoregulatory role under drought in all genotypes, they are not the main factor responsible for the differences among the genotypes. Similarly, the proline was not a specific marker for drought tolerance when the drought response of two wild wheat [*Triticum turgidum* ssp. *diccocoides* (Korn.) Thell.] genotypes were compared. The proline content was not higher in the drought tolerant cultivar (TR39477) compared to drought sensitive (TTD-22) genotype [55], in spite of the fact that elevated trascription of genes involved in proline synthesis was found in drought-tolerant genotype [56].

### Role of sugars and sugar alcohols in response to drought of the six wheat genotypes

Comparing the amount of sugars among the genotypes revealed that drought stress induced a high accumulation of sucrose together with glucose and fructose in wheat Soissons, Toborzó, Žitarka and Antonija which were grouped together according to the PCA (Fig 6). The amount of sucrose in leaf saps was extremely high (around 3mg/mL) in these genotypes. Similarly, high amount of sucrose was observed in drought-tolerant wheat genotype JD17 [17]. The amount of disaccharides 1, 2 and 3 also increased in Soissons, Toborzó and Žitarka genotypes under drought stress conditions (but not in Antonija). These results indicate that the accumulation of these osmolites can be part of an adaptation mechanism rather than degradation products in these genotypes. Similarly, high production of water soluble carbohydrates (glucose, fructose, sucrose and fructans) was also found in other drought tolerant wheat genotypes [57–59]. In Ellvis the amount of sucrose was around 2mg/mL, while this genotype possesses the lowest amount of fructose and glucose. The amount of disaccharides 1, 2 and 3 was slightly decreased. In wheat Srpanjka, the sucrose content was even lower (appr. 1mg/mL) and did not change as compared to control plants, while the fructose and glucose content was the highest in this genotype, indicating the occurrence of intense sucrose hydrolization to glucose and fructose. Similar results were found in dehydrated wheat seedling [60]. The degradation of sucrose to glucose and fructose by invertase ensured the energy necessary for respiration and carbon reserves needed for biosynthesis of other organic compounds in the cells [17, 61]. The dominance of catabolic processes in Srpanjka is also supported by the decrease of the amount of disaccharides 1, 2 and 3.

Significant correlation between the changes of osmotic potential and the amount of D1, 2, 3 and 5 disaccharides was also found (S6 Table). Besides sucrose, fructose and glucose, the accumulation of these unidentified disaccharides 1, 2, 3 and 5, could play a detrimental role in the osmotic adjustment, while others such as ribose and galactose have marginal roles. Many disaccharides, such as raffinose or threhalose are known for taking part in the osmoregulation [14]. According to their mass spectra and retention index the disaccharides found in the present study differed from raffinose and threhalose. Therefore, further investigations are planned to complete the identification of the unidentified disaccharides.

In general, sugar alcohols originate from the reduction of sugars and may have osmoprotective role [62] or may be used as translocated carbohydrates for energy and carbon supply [63]. In this experiment, the SA3 was accumulated in all genotypes under drought stress and the highest amount of SA1 and SA3 was found in Srpanjka (Fig 5). It is possible that the production of these sugar alcohols can be an alternative pathway for osmolite production in this genotype. However, further investigations are necessary to determine their roles. It was found that galactinol may be a source for production galactose and raffinose family oligosaccharides which plants may use as compatible solutes [64, 65], although the amount of galactinol decreased in a drought-sensitive wheat cultivar after drought treatment [66].

The cyclic polyol, myo-inositol was also accumulated in drought-exposed plants, but the highest production was found in Antonija followed by Žitarka which showed more negative osmotic potential values than Srpanjka (Figs 3 and 5). Correlation between the myo-inositol level and drought tolerance was also observed in chickpea [67], maize [68] and in olive tree roots [69]. It is possible that signalling the myo-inositol affects the process that protects the plants against water stress [70].

### Role of organic acids and amino acids in response to drought of the six wheat genotypes

Organic acids play important role in energy production. They are precursors of amino acids and may modulate plant adaptation to stress, including drought [71,72]. Intense accumulation of malic acid, succinic acid and galacturonic acids was identified in response to drought together with a decline of citric acid content in Bermuda grass [73]. Similarly, drought induced notable malic and oxalic acid productions in Soissons, Žitarka, Toborzó and Antonija. In the drought sensitive genotypes, especially in Ellvis, the drought had either no effect on the content of TCA cycle intermediates or decreased their accumulation levels, including the inorganic acids (Fig 5), indicating that the energy production is blocked under drought in Ellvis. These results suggest that the malic and oxalic acids have important roles in response to drought in wheat and their levels can be related to drought tolerance. Besides being an essential storage carbon molecule during drought, malate has also a notable role in pH balancing and stomatal functioning [74]. It has been reported that the malate accumulation in guard cells plays a pivotal role in osmotic adjustment [75]. A link between stomatal opening and malate accumulation in guard cells has also been demonstrated [76, 77]. Under drought, malate can be converted into starch in order to decrease osmotic potential and turgor decline, thus maintaining the cellular volume and preventing the stomata closure [78, 79]. In the present study, the correlation found between the photosynthetic activity (Pn), stomatal conductance (gs) and the production of malic acid (S6 Table) also supported the theory related to the relationship between the stomata movement and malic acid content. It is possible that stomatal closure in Ellvis can be partly due to the low malic acid levels (Fig 5). However, it should also be mentioned that high amount of sucrose can also stimulate the stomatal opening [80] and promote the photosynthesis [81, 82]. Besides malate, high amount of sucrose was found in Soissons, Žitarka, Toborzó and Antonija, which can also contribute to the higher stomatal conductance and better photosynthesis.

The TCA-*cycle* regulates the synthesis of several amino acids [83]. Higher amount of glutamic acid, GABA and threonine was found in Soissons, Žitarka, Toborzó and Antonija under drought as compared to Srpanjka and Ellvis. Glutamate derived from 2-oxoglutaric acid (an intermediate of TCA cycle) is a precursor molecule of GABA [84]. Therefore, it is reasonable that the amount of glutamate and GABA changed similarly. In a transgenic perennial grass, Creeping bentgrass, an activation of glutamate metabolic pathway via the TCA cycle also promoted the GABA production and induced the drought tolerance [23]. On the other hand, low glutamic acid level was also found in drought-sensitive wheat genotype Bahar [15]. Threonine can be accumulated through an interaction between the TCA cycle (via oxalic acid) and aspartate metabolic pathway [85, 86]. Since drought induced the accumulation of oxalic acid in Soissons, Žitarka, Toborzó and Antonija, but not in Srpanjka and Ellvis, it is possible that the elevated amount of threonine was due to the induction of aspartate metabolic pathway. This association was observed in drought-treated soybean [87] and maize [68].

### Metabolites related to oxidative damage of leaves

In conditions of reduced CO_2_ assimilation, the formation of reactive oxygen species (ROS) can be induced, which leads to oxidative damage of biomembranes [88]. A positive correlation was found between the drought-induced accumulation of MDA content and the amount of palmitic (0.694**) and stearic acids (0.702**), indicating that membrane damage is related to the elevated levels of fatty acids in leaf saps. (S7 Table). Decreased desaturation degree of fatty acids and galactolipids as well as increased phospholipid content provide a membrane stability under drought [89, 90]. Conversion of stearic acid into polyunsaturated fatty acids under drought was noticed in fenugreek (*Trigonella foenum*-*graecum* L.) genotypes [91]. However, there is a limited number of studies which demonstrate a connection between composition and saturation degree of specific fatty acid and membrane damage under water deficit. Nevertheless, since only slight differences were found either in MDA or in the palmitic and stearic acid contents among the genotypes, these results suggested that these metabolites have marginal role in drought tolerance mechanism. Our results may draw the attention to the propanoic acid, which shows negative correlation with drought stress response. It is possible that it is a precursor in the drought-induced metabolic changes, however, its role has not been investigated yet.

In addition, Soissons genotype had a slightly higher amount of galactonic acid under drought than those in Srpanjka and Ellvis (Fig 5). The galactonic acid, the oxidized form of D-galactose [92] can be an intermediate in the ascorbic acid pathway [93]. Ascorbic acid is an important antioxidant in plants against oxidative stress under stress condition [94]. In addition, galactonic acid, is a sugar acid component of pectin polysaccharides. It was presented that pectins tend to form hydrated cloaks to protect cells from dehydration [95]. Studies of wheat cultivars revealed a positive connection between pectin polymers biosynthesis and drought tolerance [96, 97]. Thus it is possible that the increase in galactonic acid in Soissons appeared to be linked with the reorganisation of cell wall polysaccharide network and drought tolerance mechanism; however, further investigations are necessary to prove it.

## Conclusions

In conclusion, in all tested genotypes the drought stress caused reduction in RWC, alteration of membrane integrity, inhibition of photosynthetic parameters (Pn, gs, Ci and E), decrease in osmotic potential and increase in accumulation levels of many compatible solutes such as sugars, sugar alcohols, organic and amino acids. Specific differences in physiological and metabolic responses among genotypes existed. Among the genotypes, Soissons proved to be the most drought tolerant cultivar able to successfully withstand the dehydration provoked by drought and to maintain the high RWC, assimilation rate and stomata conductions. Considering all physiological and metabolic responses together, Ellvis and Srpanjka were the most sensitive to drought stress. These genotypes generally had inhibited TCA*-cycle*, glycoses and amino acid metabolism, however, Srpanjka had a great accumulation of glucose and fructose, higher amount of sugar alcohols 2 and 3 and increased citric acid than Ellvis suggesting enhanced glycolysis process. The metabolic responses under drought among Soissons, Žitarka, Antonija and Toborzó were similar but varied in magnitude. They had higher TCA*-cycle* derived intermediates (malic and oxalic acid), unknown organic acid 1, higher sucrose, GABA, unidentified disaccharide 5, glutamic acid and L-threonine amount. Soissons also exhibited increased galactonic and phosphoric acid amounts. The results suggested that Soissons, Žitarka, Antonija and Toborzó genotypes showed active metabolic pathways, energy balance and carbon circulation referring to greater tendency to overcome water deficit and have a larger potential to resist drought stress than Ellvis or Srpanjka. Present results suggest that although generally used wheat genotypes have a relatively narrow genetic variation, they may use different metabolic strategies to adapt to water stress conditions. This may provide valuable information for the future breeding programmes.

## Supporting information

S1 Table Analysis variance of various physiological parameters by using factorial ANOVA in STATISTICA 13.4 software package. Mean squares followed by asterisks (*) are significantly different (P<0.05). Analyse included four repetitions for RWC and five for MDA and Photosynthetic activity (Pn), stomatal conductance (gs), intercellular CO2 level (Ci) and transpiration (E) measurements.
(DOCX)

S2 Table Analysis of variance of osmotic potential, proline and glycine-betaine by using factorial ANOVA in STATISTICA 13.4 software package. Mean squares followed by asterisks (*) are significantly different (P<0.05). Analyse included three repetitions for each parameter.
(DOCX)

S3 Table **Accumulation of soluble sugars (A), organic acids (B), sugar alcohols (C), amino acids (D) and fatty acids (E) in leaves of six wheat genotypes under control and drought.** Values are means of three repetitions per treatment ± S.D. The amounts were calculated according to the internal standard and an authentic standard or were estimated by the use of a standard from the same compound class (based on their mass spectra) having the closest tentative molecular weight. Tukey’s *post hoc* test was used to compare the mean values within each measured parameter. The different letters indicate statistically significant differences at P<0.05. D: unidentified disaccharides 1–5; OA: unidentified organic acid (1–3), SA: sugar alcohols (1–2).
(DOCX)

S4 Table Analysis of variance of sugars by using factorial ANOVA in STATISTICA 13.4 software package. Mean squares followed by asterisks (*) are significantly different (P<0.05). Unknown disaccharides (1–5) (D1, D2, D3, D4 and D5). Analyse included three repetitions for each parameter.
(DOCX)

S5 Table Analysis of variance of organic acids by using factorial ANOVA in STATISTICA 13.4 software package. Mean squares followed by asterisks (*) are significantly different (P<0.05). Organic acid (1–2) (OA1, OA2). Analyse included three repetitions for each parameter.
(DOCX)

S6 Table Analysis of variance of fatty and amino acids and sugar alcohols by using factorial ANOVA in STATISTICA 13.4 software package. Mean squares followed by asterisks (*) are significantly different (P<0.05). Unknown sugar alcohols (1–3) (SA1, SA2 and SA3). Analyse included three repetitions for each parameter.
(DOCX)

S7 Table The results of correlation analyses between different physiological and metabolic parameters. Spearmanʾs correlation coefficients (R) were calculated for control and drought using STATISTICA 13.4 software package. Correlations are significant at P<0.05 (*) and (**) at P<0.01, respectively. Analyse included three repetitions for each parameter.
(DOCX)

S8 Table Factor loadings of metabolic compounds, proline, GB and osmotic potential of six wheat genotypes. Principle component analysis (PCA) was applied for evaluation of metabolic response of wheat genotypes under control and drought. Data were analysed by using STATISTICA 13.4 software package.
(DOCX)

S1 Fig Principal component analysis of combined wheat genotypes data sets. Loadings of the first two factors. Colour of metabolites and osmolytes: sugars (red), organic acids (violet), amino acids (green), sugar alcohols (orange), fatty acids (blue), osmotic potential (black), proline (brown) and GB (olive green). Principle component analysis (PCA) was applied for evaluation of metabolic response of wheat genotypes under control and drought. Data were analysed by using STATISTICA 13.4 software package.
(TIF)
