# Supplementary material for: Metabolic response to drought in six winter wheat genotypes
Source: PLoS One. 2019 Feb 19;14(2):e0212411. doi: 10.1371/journal.pone.0212411 (PMC6380608; doi:10.1371/journal.pone.0212411)
Supplement: S1 Table — Mean squares followed by asterisks (*) are significantly different (P<0.05). Analyse included four repetitions for RWC and five for MDA and Photosynthetic activity (Pn), stomatal conductance (gs), intercellular CO2 level (Ci) and transpiration (E) measurements. (DOCX) [file pone.0212411.s001.docx]

| Source of variation | Df | MS | | | | | |
| --- | --- | --- | --- | --- | --- | --- | --- |
|  |  | RWC | Pn | gs | Ci | E | MDA |
| Genotype (G) | 5 | 78.36* | 2.87* | 2088.26* | 165.58 ns | 0.20* | 11.89* |
| Treatment (T) | 1 | 2451.26* | 900.94* | 750457.58* | 32806.82* | 102.97* | 191.80* |
| G*T | 5 | 63.40* | 6.51* | 1177.03* | 125.98 ns | 0.15* | 1.47ns |
|  |  |  |  |  |  |  |  |

*significant at P≤0.05; ns-not significant

Error: RWC (17.60), Pn (0.64), gs (150.87), Ci (199.95), E (0.03), MDA (2.43)
