# Supplementary material for: Metabolic response to drought in six winter wheat genotypes
Source: PLoS One. 2019 Feb 19;14(2):e0212411. doi: 10.1371/journal.pone.0212411 (PMC6380608; doi:10.1371/journal.pone.0212411)
Supplement: S2 Table — Mean squares followed by asterisks (*) are significantly different (P<0.05). Analyse included three repetitions for each parameter. (DOCX) [file pone.0212411.s002.docx]

| Source of variation | Df | MS | | |
| --- | --- | --- | --- | --- |
|  |  | Ψπ | Pro | GB |
| Genotype (G) | 5 | 0.06* | 88.72* | 5163.81* |
| Treatment (T) | 1 | 3.56* | 26382.81* | 27660.67* |
| G*T | 5 | 0.05* | 92.18* | 4162.14* |
|  |  |  |  |  |

*significant at P≤0.05; ns-not significant

Error: Ψπ (0.01), Pro (15.19), GB (175.01)
