# Supplementary material for: Metabolic response to drought in six winter wheat genotypes
Source: PLoS One. 2019 Feb 19;14(2):e0212411. doi: 10.1371/journal.pone.0212411 (PMC6380608; doi:10.1371/journal.pone.0212411)
Supplement: S3 Table — Accumulation of soluble sugars (A), organic acids (B), sugar alcohols (C), amino acids (D) and fatty acids (E) in leaves of six wheat genotypes under control and drought. Values are means of three repetitions per treatment ± S.D. The amounts were calculated according to the internal standard and an authentic standard or were estimated by the use of a standard from the same compound class (based on their mass spectra) having the closest tentative molecular weight. Tukey’s post hoc test was used to compare the mean values within each measured parameter. The different letters indicate statistically significant differences at P<0.05. D: unidentified disaccharides 1–5; OA: unidentified organic acid (1–3), SA: sugar alcohols (1–2). (DOCX) [file pone.0212411.s003.docx]

**S3 Table. Accumulation of soluble sugars (A), organic acids (B), sugar alcohols (C), amino acids (D) and fatty acids (E) in leaves of six wheat genotypes under control and drought.**

|  |  | **Soissons** | | **Žitarka** | | **Srpanjka** | | **Antonija** | | **Toborzó** | | **Ellvis** | |
| --- | --- | --- | --- | --- | --- | --- | --- | --- | --- | --- | --- | --- | --- |
|  |  | **Control** | **Drought** | **Control** | **Drought** | **Control** | **Drought** | **Control** | **Drought** | **Control** | **Drought** | **Control** | **Drought** |
| Sucrose | **Ribose** | 23.6  ±3.7  a | 21.8  ±3.6  a | 17.6  ±3.0  a | 20.8  ±2.6  a | 19.7  ±2.2  a | 18.0  ±2.8  a | 18.4  ±3.1  a | 21.7  ±3.6  a | 20.0  ±4.5  a | 23.5  ±3.6  a | 19.8  ±2.1  a | 20.3  ±2.5  a |
|  | **Glucose** | 733  ±126  def | 1089  ±106  c | 526  ±71  ef | 1852  ±191  a | 658  ±209  def | 1917  ±137  a | 398  ±96  f | 1287  ±69  b | 450  ±100  ef | 1170  ±157  bc | 407  ±86  f | 970  ±110  cd |
|  | **Fructose** | 662  ±97  bcd | 726  ±73  bc | 418  ±80  efg | 874  ±65  b | 564  ±132  cde | 1584  ±130  a | 269  ±49  g | 538  ±39  cde | 333  ±85  efg | 817  ±66  b | 305  ±93  g | 441  ±67  defg |
|  | **Galactose** | 15.3  ±3.1  de | 19.0  ±5.7  cde | 8.3  ±2.2  f | 28.3  ±2.1  abc | 9.8  ±3.0  ef | 31.0  ±4.5  ab | 5.3  ±1.4  f | 21.1  ±3  bcd | 7.9  ±3.2  f | 27.5  ±3.1  abc | 6.4  ±1.4  f | 33.9  ±5.6  a |
|  | **Sucrose** | 6.2  ±1.1  f | 3095  ±439  ab | 46.6  ±18  f | 2965  ±217  ab | 1096  ±369  de | 1129  ±34  de | 1346  ±298  d | 3693  ±285  a | 359  ±76  ef | 2863  ±239  bc | 886  ±94  de | 2150  ±302  c |
|  | **D1** | 1.1  ±1.0  f | 164  ±20  b | 16.5  ±6.5  e | 108  ±7.4  cd | 217  ±24  a | 89  ±3.4  de | 209  ±22  a | 106  ±12  cd | 88.5  ±12  d | 162  ±18  b | 142  ±14  bc | 149  ±17  bc |
|  | **D2** | 1.6  ±1.0  e | 74.4  ±14  b | 5.5  ±3.2  e | 36.9  ±3.4  d | 137  ±17  a | 44  ±2.2  cd | 138  ±14  a | 31  ±3.2  d | 50.3  ±6.1  c | 74.5  ±12  b | 88.6  ±6.0  b | 77.3  ±10  b |
|  | **D3** | 1.9  ±1.8  g | 119  ±34  cd | 25.8  ±8.2  ef | 37.5  ±6.1  e | 322  ±42  a | 91.5  ±5.0  d | 329  ±31  a | 21.5  ±3.2  f | 120  ±14  c | 123  ±24  c | 222  ±16  b | 155  ±23  c |
|  | **D4** | 0.7  ±0,2  f | 1.4  ±1.0  f | 1.3  ±0.9  f | 0.5  ±0.4  f | 12.8  ±1.4  b | 29.9  ±2.3  a | 10.7  ±1.0  b | 0.8  ±0.5  f | 6.1  ±1.2  d | 1.8  ±0.8  ef | 8.3  ±0.6  c | 4.1  ±0.9  de |
|  | **D5** | 0.6  ±0.4  f | 59.4  ±6.3  ab | 1.9  ±1.4  f | 47.9  ±3.3  b | 30.1  ±5.3  c | 29.9  ±2.2  cd | 34.1  ±3.2  c | 48.3  ±4.6  b | 10.9  ±1.6  e | 59.9  ±3.2  a | 23.0  ±2.0  d | 49.7  ±4.4  b |
| organic acids | **cis-aconitic acids** | 456  ±60  ab | 424  ±52  ab | 349  ±44  bc | 513  ±38  ab | 249  ±59  b | 232  ±62  b | 334  ±41  bc | 467  ±52  ab | 457  ±144  ab | 420  ±75  ab | 557  ±80  a | 558  ±93  a |
|  | **Malic acid** | 124  ±28  cd | 222  ±37  ab | 105  ±19  d | 193  ±39  b | 158  ±9.1  bc | 201  ±12  bc | 124  ±22  d | 263  ±27  a | 111  ±29  d | 191  ±11  bc | 92  ±12  d | 85  ±9.2  d |
|  | **Phosphoric**  **acid** | 99  ±29  abc | 137  ±10  a | 71  ±13  c | 100  ±7.6  b | 54  ±12  cd | 40  ±4.1  d | 68  ±15  c | 104  ±8.9  b | 105  ±30  ab | 111  ±17  ab | 76  ±12  c | 55  ±7.1  d |
|  | **Galactonic acid** | 22.2  ±3.1  abc | 25.2  ±4.1  a | 17.4  ±4.0  ab | 19.7  ±2.4  ab | 17.2  ±3.1  ab | 15.2  ±2.4  b | 16.9  ±4.0  b | 18.9  ±3.2  ab | 18.8  ±5.2  ab | 22.3  ±3.1  ab | 15.9  ±2.1  b | 15.0  ±1.8  b |
|  | **Citric acid** | 71  ±21  cd | 65  ±18  cd | 77  ±11  cd | 99  ±28  c | 341  ±48  a | 342  ±27  a | 201  ±42  b | 160  ±32  b | 198  ±134  b | 64  ±13  de | 184  ±19  b | 43  ±5  e |
|  | **Succinic acid** | 5.2  ±1.4  a | 5.1  ±1.3  a | 4.9  ±0.8  a | 5.3  ±0.5  a | 6.0  ±0.7  a | 4.4  ±0.4  a | 5.9  ±0.9  a | 6.4  ±0.8  a | 6.3  ±1.5  a | 5.1  ±0.6  a | 6.0  ±0.8  a | 5.0  ±0.9  ab |
|  | **Oxalic acid** | 5.9  ±1.7  c | 22.9  ±2.7  a | 7.2  ±1.1  c | 23.2  ±2.6  a | 7.3  ±2.8  cd | 4.5  ±0.8  d | 10.0  ±2.8  c | 21.6  ±1.9  a | 16.3  ±8.4  b | 21.6  ±2  a | 19.5  ±3.2  ab | 19.2  ±2.8  ab |
|  | **OA1** | 5.6  ±1.4  ab | 7.2  ±0.6  a | 5.6  ±1.2  ab | 6.7  ±1.5  ab | 7.9  ±1.4  a | 5.8  ±0.9  b | 3.3  ±0.7  cd | 5.8  ±0.8  b | 3.8  ±0.7  c | 7.5  ±1.5  ab | 2.5  ±0.6  d | 4.1  ±0.8  c |
|  | **OA2** | 2.9  ±1.7  d | 16.0  ±2.4  a | 6.8  ±0.8  c | 19.3  ±2.7  a | 5.9  ±2.6  cd | 0.1  ±0  e | 7.1  ±1.8  cd | 11.2  ±0.8  bc | 14.5  ±2.8  ab | 8.6  ±2.5  c | 15.9  ±1.4  a | 5.3  ±0.8  d |
|  | | | | | | | | | | | | | |
| continue | | | | | | | | | | | | | |
|  |  | **Soissons** | | **Žitarka** | | **Srpanjka** | | **Antonija** | | **Toborzó** | | **Ellvis** | |
|  |  | **Control** | **Drought** | **Control** | **Drought** | **Control** | **Drought** | **Control** | **Drought** | **Control** | **Drought** | **Control** | **Drought** |
| sugar alcohols | **SA1** | 8.7  ±2.7  cde | 11.6  ±2.5  bc | 10.7  ±1.8  bc | 11.6  ±1.2  bc | 18.8  ±1,5  a | 17.7  ±1.3  a | 10.0  ±1.2cccd | 11.6  ±1.5  bc | 6.4  ±1.3  e | 12.8  ±2.7  bc | 7.6  ±1.4  de | 13.4  ±1.2  b |
|  | **SA2** | 10.5  ±2.4  a | 3.3  ±0.8  cd | 3.4  ±0.7  cd | 5.8  ±0.8  b | 3.7  ±1.6  cd | 9.0  ±1.2  a | 0.1  ±0  e | 3.0  ±0.7  cd | 1.9  ±1.2  d | 4.8  ±1.0  bc | 0.1  ±0  e | 4.5  ±1.2  bc |
|  | **SA3** | 3.2  ±3.3  e | 22.1  ±2.8  bc | 10.6  ±4.5  de | 25.0  ±5.9  b | 26.4  ±3.5  ab | 32.1  ±2.9  a | 17.5  ±4.7  bcd | 25.0  ±1.2  b | 10.7  ±0.9  de | 23.3  ±4.4  bc | 13.5  ±2.8  cde | 19.2  ±1.9  bcd |
|  | **Myo-inositol** | 2.3  ±0.9  f | 24.2  ±3.0  c | 5.3  ±0.8  f | 28.0  ±3.9  b | 13.7  ±2.1  d | 21.0  ±0.1  c | 12.7  ±2.9  d | 37.0  ±3.6  a | 11.7  ±4.2  d | 25.6  ±3.1  c | 12.4  ±2,6  d | 21.2  ±1.8  c |
| Amino acid | **Glutamic acid** | 0.1  ±0.  f | 3.8  ±0.9  bc | 0.1  ±0.05  f | 6.5  ±1.1  b | 1.2  ±0.3  e | 1.9  ±0.4  de | 2.3  ±0.4  d | 10.2  ±0.9  a | 1.0  ±0.1  e | 3.7  ±0.2  c | 1.6  ±0.3  de | 0.9  ±0.3  e |
|  | **GABA** | 6.8  ±1.9  d | 36.8  ±8.1  a | 6.4  ±4.8  d | 28.5  ±5.2  ab | 8.1  ±3.8  cd | 21.9  ±4.2  b | 6.7  ±0.5  d | 36.8  ±6.1  a | 9.3  ±3.9  c | 29.9  ±3.0  a | 6.2  ±1.4  d | 13.8  ±2.0  c |
|  | **L-Threonin** | 0.1  ±0.1  e | 38.7  ±2.4  b | 0.1  ±0.1  e | 45.6  ±2.0  a | 0.1  ±0.1  d | 20.6  ±1.4  d | 0.1  ±0.1  e | 42.2  ±2.8  ab | 0.1  ±0.1  e | 36.6  ±2.9  b | 0.1  ±0.1  e | 27.1  ±1.3  c |
| Lipids | **Stearic acid** | 0.1  ±0.08  e | 25.1  ±1.6  c | 0.1  ±0.07  e | 32.0  ±1.9  b | 0.1  ±0.1  e | 12.2  ±0.5  d | 0.1  ±0.07  e | 38.4  ±1.9  a | 0.1  ±0.09  e | 23.0  ±1.8  c | 0.1  ±0.1  e | 14.4  ±1.0  d |
|  | **Palmitic acid** | 7.40  ±1.3  f | 31.8  ±2.8  ab | 12.1  ±0.9  e | 36.9  ±3.1  ab | 17.7  ±3.8  de | 23.4  ±2.3  c | 15.6  ±2.3  d | 38.5  ±2.8  a | 20.3  ±3.2  cd | 30.2  ±3.9  b | 16.2  ±1.6  d | 28.3  ±2.7  b |
|  | **Propanoic acid** | 14.7  ±2.9  ab | 7.9  ±1.4  cd | 8.4  ±1.7  cd | 4.5  ±0.8  e | 12.4  ±2.7  bc | 4.1  ±0.7  e | 10.4  ±0.9  c | 5.1  ±1.2  e | 16.1  ±2.0  ab | 7.7  ±1.4  d | 17.9  ±3.1  a | 4.1  ±1.0  e |
