# Supplementary material for: Metabolic response to drought in six winter wheat genotypes
Source: PLoS One. 2019 Feb 19;14(2):e0212411. doi: 10.1371/journal.pone.0212411 (PMC6380608; doi:10.1371/journal.pone.0212411)
Supplement: S4 Table — Mean squares followed by asterisks (*) are significantly different (P<0.05). Unknown disaccharides (1–5) (D1, D2, D3, D4 and D5). Analyse included three repetitions for each parameter. (DOCX) [file pone.0212411.s004.docx]

| Source of variation | Df | Sugars | | | | | | | | | |
| --- | --- | --- | --- | --- | --- | --- | --- | --- | --- | --- | --- |
|  |  | Ribose | Fructose | Glucose | Galactose | Sucrose | D1 | D2 | D3 | D4 | D5 |
| Genotype (G) | 5 | 0.00 ns | 55.98* | 46.26* | 0.002* | 248.53* | 1.84* | 0.93* | 5.92* | 0.01* | 0.04* |
| Treatment (T) | 1 | 0.00 ns | 235.34* | 774.18* | 0.16* | 7088.05* | 0.51ns | 0.32* | 10.64* | 0.04* | 1.79* |
| G*T | 5 | 0.00 ns | 25.78* | 43.15* | 0.01* | 359.02* | 3.68* | 1.49* | 7.43* | 0.01* | 0.15* |

*significant at P≤0.05; ns-not significant

Error (sugars): ribose, galactose, D4 (0.00), 1.04 (fructose), glucose (2.18), sucrose (12.62), D1 (0.12), D2 (0.06), D3 (0.32), D5 (0.01)
