# Supplementary material for: Metabolic response to drought in six winter wheat genotypes
Source: PLoS One. 2019 Feb 19;14(2):e0212411. doi: 10.1371/journal.pone.0212411 (PMC6380608; doi:10.1371/journal.pone.0212411)
Supplement: S5 Table — Mean squares followed by asterisks (*) are significantly different (P<0.05). Organic acid (1–2) (OA1, OA2). Analyse included three repetitions for each parameter. (DOCX) [file pone.0212411.s005.docx]

| Source of variation | Df | Organic acids | | | | | | | | |
| --- | --- | --- | --- | --- | --- | --- | --- | --- | --- | --- |
|  |  | *cis*-aconitic | Malic | Phosphoric | Galactonic | Citric | Succinic | Oxalic | OA1 | OA2 |
| Genotype (G) | 5 | 34.43* | 5.08* | 2.40* | 0.03* | 1.66* | 0.0001ns | 0.002* | 0.039* | 0.006* |
| Treatment (T) | 1 | 6.77ns | 29.51* | 0.795ns | 0.009ns | 0.70* | 0.001ns | 0.009* | 0.008ns | 0.010* |
| G*T | 5 | 7.42ns | 2.29* | 0.607ns | 0.004ns | 0.24* | 0.001ns | 0.002* | 0.095* | 0.003* |

* significant at P≤0.05; ns-not significant

Error: cis-aconitic acid (4.08), malic acid (0.39), phosphoric acid (0.24), galactonic acid (0.01), citric acid (0.07), succinic acid and OA2 (0.001), oxalic acid (0.000) and OA1 (0.013)
