# Supplementary material for: Metabolic response to drought in six winter wheat genotypes
Source: PLoS One. 2019 Feb 19;14(2):e0212411. doi: 10.1371/journal.pone.0212411 (PMC6380608; doi:10.1371/journal.pone.0212411)
Supplement: S6 Table — Mean squares followed by asterisks (*) are significantly different (P<0.05). Unknown sugar alcohols (1–3) (SA1, SA2 and SA3). Analyse included three repetitions for each parameter. (DOCX) [file pone.0212411.s006.docx]

| Source of variation | Df | Fatty acids | | | Amino acids | | | Sugar alcohols | | | |
| --- | --- | --- | --- | --- | --- | --- | --- | --- | --- | --- | --- |
|  |  | Stearic | Palmitic | Propanoic | Glutamic acid | GABA | L-threonine 1 | SA1 | SA2 | SA3 | Myo-inositol 6TMS |
| Genotype (G) | 5 | 0.004* | 0.001* | 0.019* | 0.004* | 0.077* | 0.007* | 0.033* | 0.017* | 0.11* | 0.063* |
| Treatment (T) | 1 | 0.141* | 0.067* | 0.325* | 0.020* | 2.321* | 0.650* | 0.041* | 0.018* | 0.635* | 1.825* |
| G*T | 5 | 0.004* | 0.003* | 0.011ns | 0.003* | 0.072* | 0.007* | 0.007* | 0.019* | 0.026* | 0.048* |

* significant at P≤0.05; ns-not significant

Error (fatty acids): stearic (0.0003), palmitic (0.0004), propanoic (0.0043)

Error (amino acids): glutamic acid (0.0002), GABA (0.0174), SA3 (0.009), L-threonine 1 (0.0010)

Error (sugar alcohols): SA1 (0.002), SA2 (0.001), SA3 (0.009), myo-inositol (0.007)
