# Supplementary material for: Metabolic response to drought in six winter wheat genotypes
Source: PLoS One. 2019 Feb 19;14(2):e0212411. doi: 10.1371/journal.pone.0212411 (PMC6380608; doi:10.1371/journal.pone.0212411)
Supplement: S8 Table — Principle component analysis (PCA) was applied for evaluation of metabolic response of wheat genotypes under control and drought. Data were analysed by using STATISTICA 13.4 software package. (DOCX) [file pone.0212411.s008.docx]

| Variable | PC-1 | PC-2 | PC-3 |
| --- | --- | --- | --- |
| Ribose | -0.470 | 0.376 | 0.111 |
| Fructose | -0.494 | -0.718 | 0.236 |
| Glucose | -0.754 | -0.490 | 0.168 |
| Galactose | -0.795 | -0.367 | 0.180 |
| Sucrose | -0.916 | 0.206 | -0.309 |
| D1 | -0.146 | 0.037 | -0.940 |
| D2 | 0.174 | -0.022 | -0.925 |
| D3 | 0.404 | -0.057 | -0.866 |
| D4 | 0.604 | -0.081 | -0.774 |
| D5 | -0.828 | 0.079 | -0.485 |
| cis-aconitic a. | -0.214 | 0.751 | 0.199 |
| Malic a. | -0.789 | -0.127 | -0.071 |
| Phosphoric a. | -0.415 | 0.715 | 0.268 |
| Galactonic a. | -0.377 | 0.415 | 0.354 |
| Citric a. | 0.298 | -0.565 | -0.492 |
| Succinic a. | 0.241 | 0.602 | -0.473 |
| Oxalic a. | -0.647 | 0.681 | -0.211 |
| OA1 | -0.267 | 0.748 | -0.169 |
| OA2 | -0.526 | -0.331 | 0.083 |
| SA1 | -0.306 | -0.820 | -0.336 |
| SA2 | -0.242 | -0.556 | 0.671 |
| SA3 | -0.650 | -0.511 | -0.524 |
| Myo-inositol | -0.914 | 0.028 | -0.302 |
| Glutamic a. | -0.762 | 0.249 | -0.179 |
| GABA | -0.953 | 0.081 | -0.013 |
| L-Threonin | -0.993 | 0.071 | 0.003 |
| Stearic a. | -0.969 | 0.131 | -0.003 |
| Palmitic a. | -0.929 | 0.141 | -0.229 |
| Propanoic a. | 0.760 | 0.431 | -0.013 |
| Pro | -0.956 | -0.122 | 0.017 |
| GB | -0.769 | 0.105 | 0.050 |
| O.P. | -0.659 | -0.061 | -0.143 |
| Explained variance (eigenvalue) | 13.868 | 5.742 | 5.372 |
| Proportion of total variance (%) | 43.338 | 17.944 | 16.788 |
| Cumulative variance (%) | 43.338 | 61.282 | 78.070 |

PC-1 (principal component 1); PC-2 (principal component 2); PC-3 (principal component 3)
