# Supplementary figures and images for: Metabolic response to drought in six winter wheat genotypes
Source: PLoS One. 2019 Feb 19;14(2):e0212411. doi: 10.1371/journal.pone.0212411 (PMC6380608; doi:10.1371/journal.pone.0212411)

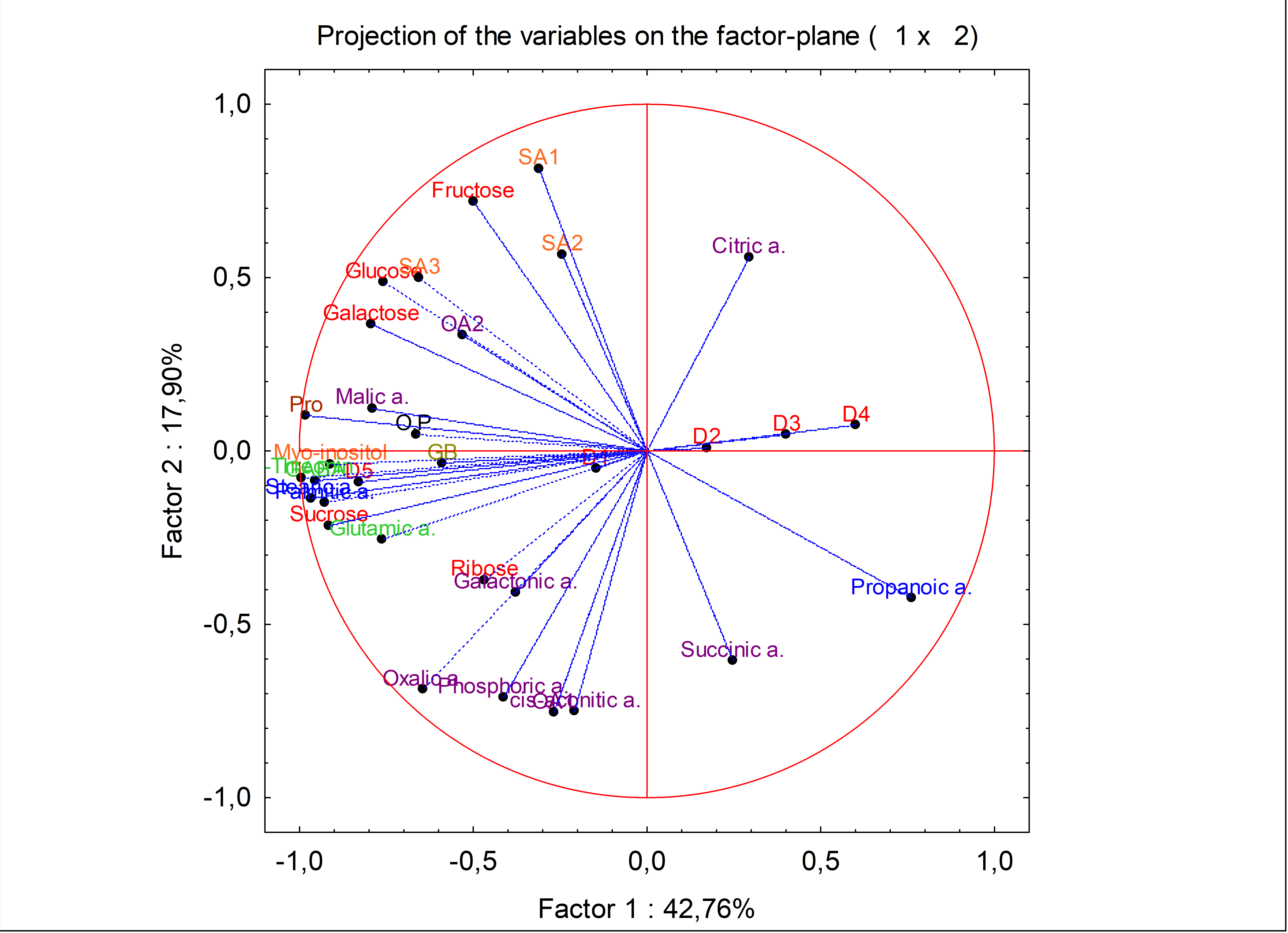

Supplement: S1 Fig — Loadings of the first two factors. Colour of metabolites and osmolytes: sugars (red), organic acids (violet), amino acids (green), sugar alcohols (orange), fatty acids (blue), osmotic potential (black), proline (brown) and GB (olive green). Principle component analysis (PCA) was applied for evaluation of metabolic response of wheat genotypes under control and drought. Data were analysed by using STATISTICA 13.4 software package. (TIF) [file pone.0212411.s009.tif]
