# Supplementary material for: Metabolic response to drought in six winter wheat genotypes
Source: PLoS One. 2019 Feb 19;14(2):e0212411. doi: 10.1371/journal.pone.0212411 (PMC6380608; doi:10.1371/journal.pone.0212411)
Supplement: Supplementary file 9 [file pone.0212411.s007.docx]

Correlations

	ribose	fructose	glucose	galactose	sucrose	D1	D2	D3	D4	D5	cisacon	malic	foszforic	galactonic	citric	succinic	oxalic	OA1	OA2	SA1	SA2	SA3	Myoinoz	Glua	GABA	Threo	Stearic	Palmitic	propanoid	Prolin	GB	YP
ribose	-			*(+)	*(+)						**(+)	*(+)	**(+)	**(+)				*(+)					*(+)		*(+)	*(+)	*(+)	*(+)				
fructose	0,095	-	**(+)	**(+)				*(-)	*(-)		*(-)	**(+)				*(-)			*(+)	**(+)	**(+)	**(+)	*(+)		**(+)	*(+)	*(+)		**(-)	**(+)	**(+)	
glucose	0,155	0,835	-	**(+)	**(+)		*(-)	**(-)	**(-)	*(+)		**(+)								**(+)	**(+)	**(+)	**(+)	**(+)	**(+)	**(+)	**(+)	**(+)	**(-)	**(+)	**(+)	**(+)
galactose	0,344	0,658	0,815	-	**(+)			*(-)	**(-)	**(+)		**(+)				*(-)				*(+)	**(+)	**(+)	**(+)	*(+)	**(+)	**(+)	**(+)	**(+)	**(-)	**(+)	**(+)	**(+)
sucrose	0,338	0,181	0,489	0,549	-	**(+)				**(+)		**(+)	*(+)					*(+)				**(+)	**(+)	**(+)	**(+)	**(+)	**(+)	**(+)	**(-)	**(+)		**(+)
D1	0,088	-0,166	-0,140	-0,044	0,444	1,000	**(+)	**(+)	**(+)	**(+)											*(-)	**(+)										
D2	-0,047	-0,269	-0,337	-0,258	0,148	0,946	-	**(+)	**(+)	*(+)					*(+)						*(-)											
D3	-0,137	-0,340	-0,461	-0,405	-0,083	0,845	0,970	-	**(+)				*(-)		**(+)						*(-)				*(-)	*(-)	**(-)					
D4	-0,205	-0,389	-0,530	-0,544	-0,314	0,678	0,855	0,942	-			*(-)	*(-)	*(-)	**(+)	*(+)					*(-)			*(-)	**(-)	**(-)	**(-)	*(-)	**(+)	**(-)		**(-)
D5	0,309	0,209	0,402	0,555	0,925	0,666	0,406	0,182	-0,084	-		**(+)										**(+)	**(+)	**(+)	**(+)	**(+)	**(+)	**(+)	**(-)	**(+)		**(+)
cisacon	0,419	-0,299	-0,008	0,156	0,310	0,065	-0,047	-0,101	-0,144	0,261	-		**(+)		**(-)		*(+)	**(+)		**(-)												*(+)
malic	0,371	0,478	0,588	0,418	0,708	0,195	-0,049	-0,215	-0,317	0,588	0,138	-	**(+)	**(+)					*(+)			**(+)	**(+)	**(+)	**(+)	**(+)	**(+)	**(+)	**(-)	**(+)	**(+)	**(+)
foszforic	0,437	-0,118	-0,003	0,019	0,452	0,065	-0,103	-0,223	-0,303	0,349	0,614	0,544	-	**(+)	*(-)			**(+)		**(-)				*(+)	**(+)	*(+)	*(+)	*(+)				
galactonic	0,547	0,079	0,091	0,153	0,344	0,129	-0,017	-0,107	-0,186	0,327	0,479	0,607	0,753	-				*(+)	*(+)						**(+)							
citric	-0,110	0,273	0,102	-0,173	-0,212	0,317	0,408	0,470	0,589	-0,121	-0,299	0,234	-0,250	-0,031	-					**(+)		*(+)									*(+)	*(-)
succinic	0,071	-0,285	-0,174	-0,210	0,170	0,373	0,335	0,308	0,363	0,170	0,442	0,303	0,447	0,396	0,308	-																*(-)
oxalic	0,312	-0,088	-0,053	0,083	0,142	0,137	0,064	0,019	0,001	0,194	0,372	-0,096	0,191	0,026	-0,151	0,320	-	*(+)														
OA1	0,343	-0,194	0,003	-0,060	0,346	0,125	-0,019	-0,094	-0,121	0,263	0,433	0,229	0,516	0,409	-0,128	0,125	0,388	-			*(-)		*+	*+			*(+)	**(+)				
OA2	0,209	0,385	0,327	0,230	0,184	-0,090	-0,197	-0,252	-0,256	0,135	-0,253	0,427	0,130	0,367	0,064	0,058	0,081	-0,048	-	*+	*+	*+			*(+)							
SA1	-0,089	0,525	0,448	0,407	0,094	0,209	0,177	0,145	0,133	0,199	-0,520	0,191	-0,604	-0,282	0,421	-0,161	-0,103	-0,356	0,380	-		**+							*(-)		*(+)	
SA2	0,300	0,701	0,522	0,527	-0,133	-0,339	-0,344	-0,340	-0,331	-0,089	-0,160	0,191	-0,142	0,187	0,070	-0,252	-0,030	-0,350	0,354	0,405	-											
SA3	0,062	0,628	0,647	0,553	0,596	0,445	0,261	0,115	-0,031	0,665	-0,371	0,591	-0,216	-0,070	0,342	-0,194	0,011	0,040	0,367	0,677	0,193	-	**+	**+	**(+)	**(+)	**(+)	**(+)	**(-)	**(+)	**(+)	**(+)
Myoinoz	0,339	0,332	0,646	0,660	0,903	0,327	0,039	-0,165	-0,317	0,810	0,309	0,766	0,356	0,315	0,010	0,196	0,088	0,318	0,152	0,179	-0,063	0,666	-	**+	**(+)	**(+)	**(+)	**(+)	**(-)	**(+)	**(+)	**(+)
Glua	0,286	0,135	0,510	0,341	0,819	0,155	-0,097	-0,265	-0,370	0,587	0,245	0,767	0,415	0,271	-0,036	0,205	-0,025	0,370	0,154	-0,014	-0,172	0,431	0,822	-	**(+)	**(+)	**(+)	**(+)	*(-)	**(+)		**(+)
GABA	0,398	0,436	0,619	0,604	0,860	0,173	-0,126	-0,333	-0,507	0,754	0,243	0,856	0,553	0,510	-0,140	0,107	0,074	0,293	0,346	0,080	0,111	0,568	0,835	0,792	-	**(+)	**(+)	**(+)	**(-)	**(+)	*(+)	**(+)
Threo	0,335	0,419	0,717	0,750	0,905	0,129	-0,183	-0,401	-0,601	0,797	0,301	0,708	0,441	0,354	-0,310	-0,016	0,114	0,308	0,235	0,113	0,116	0,548	0,875	0,758	0,897	-	**(+)	**(+)	**(-)	**(+)	*(+)	**(+)
Stearic	0,344	0,349	0,673	0,652	0,911	0,087	-0,224	-0,435	-0,603	0,740	0,282	0,749	0,445	0,338	-0,269	0,042	0,106	0,372	0,268	0,084	0,036	0,550	0,903	0,845	0,877	0,959	-	**(+)	**(-)	**(+)	*(+)	**(+)
Palmitic	0,323	0,263	0,596	0,614	0,880	0,294	0,001	-0,198	-0,355	0,787	0,407	0,717	0,473	0,405	-0,085	0,174	0,147	0,482	0,141	0,078	-0,119	0,597	0,938	0,761	0,814	0,887	0,902	-	**(-)	**(+)	*(+)	**(+)
propanoid	0,151	-0,474	-0,672	-0,689	-0,556	0,019	0,211	0,347	0,474	-0,505	0,172	-0,446	0,063	0,111	0,182	0,146	0,052	0,143	-0,264	-0,384	-0,225	-0,572	-0,608	-0,416	-0,529	-0,674	-0,638	-0,549	-	**(-)	**(-)	**(-)
Prolin	0,284	0,613	0,695	0,794	0,754	0,138	-0,123	-0,312	-0,524	0,737	0,149	0,761	0,321	0,267	-0,155	-0,264	0,137	0,342	0,161	0,191	0,118	0,627	0,765	0,549	0,808	0,877	0,801	0,774	-0,656	-	**(+)	**(+)
GB	-0,105	0,740	0,692	0,582	0,286	-0,047	-0,151	-0,205	-0,232	0,252	-0,293	0,585	-0,277	-0,248	0,427	-0,186	-0,149	-0,233	0,007	0,437	0,251	0,608	0,51	0,356	0,435	0,401	0,4	0,366	-0,583	0,544	-	*(+)
YP	0,297	0,269	0,600	0,766	0,844	0,175	-0,105	-0,304	0,484	0,766	0,386	0,516	0,241	0,101	-0,345	-0,163	0,126	0,241	-0,029	0,145	-0,042	0,510	0,866	0,653	0,753	0,902	0,855	0,872	-0,625	0,846	0,483	-

*. Correlation is significant at the 0.05 level (2-tailed).

**. Correlation is significant at the 0.01 level (2-tailed).
